# Digital symbol-digit modalities test with modified flexible protocols in patients with CNS demyelinating diseases

**DOI:** 10.1038/s41598-024-65486-3

**Published:** 2024-06-25

**Authors:** Dayoung Seo, Jeong Min So, Jiyon Kim, Heejae Jung, Inhye Jang, Hyunjin Kim, Dong-Wha Kang, Young-Min Lim, Jaesoon Choi, Eun-Jae Lee

**Affiliations:** 1 AMIST, University of Ulsan College of Medicine, Seoul, 05505 South Korea; 2 Department of Neurology, Asan Medical Center, University of Ulsan, Seoul, 05505 South Korea; 3 Biomedical Engineering, University of Ulsan College of Medicine, Seoul, 05505 South Korea; 4 Translational Biomedical Research Group, Asan Medical Center, University of Ulsan, Seoul, 05505 South Korea

**Keywords:** Demyelinating diseases, Multiple sclerosis

## Abstract

Cognitive impairment (CI) is prevalent in central nervous system demyelinating diseases, such as multiple sclerosis (MS) and neuromyelitis optica spectrum disorders (NMOSD). We developed a novel tablet-based modified digital Symbol Digit Modalities Test (MD-SDMT) with adjustable protocols that feature alternating symbol-digit combinations in each trial, lasting one or two minutes. We assessed 144 patients (99 with MS and 45 with NMOSD) using both MD-SDMT protocols and the traditional paper-based SDMT. We also gathered participants’ feedback through a questionnaire regarding their preferences and perceived reliability. The results showed strong correlations between MD-SDMT and paper-based SDMT scores (Pearsons correlation: 0.88 for 2 min; 0.85 for 1 min, both p < 0.001). Among the 120 respondents, the majority preferred the digitalized SDMT (55% for the 2 min, 39% for the 1 min) over the paper-based version (6%), with the 2 min MD-SDMT reported as the most reliable test. Notably, patients with NMOSD and older individuals exhibited a preference for the paper-based test, as compared to those with MS and younger patients. In summary, even with short test durations, the digitalized SDMT effectively evaluates cognitive function in MS and NMOSD patients, and is generally preferred over the paper-based method, although preferences may vary with patient characteristics.

## Introduction

Central nervous system (CNS) inflammatory demyelinating diseases (CIDDs), including multiple sclerosis (MS) and neuromyelitis optica spectrum disorders (NMOSD), exhibit relapses or neurodegeneration throughout life, accompanied by a range of symptoms based on lesion location^1^. Cognitive function, which is an important determinant of quality of life, is one of the aspects negatively impacted by CIDDs^2^. In MS, up to 70% of patients experience varying degrees of cognitive impairment, often progressing insidiously without clear relapses^3,4^. The most commonly affected domains are information processing speed, attention, learning, and memory^5,6^. In NMOSD, patients also commonly experience cognitive impairment, with a potential of attack-independent deterioration^7^. Structural abnormalities in brain white and grey matter, along with functional alterations in strategic brain networks, may contribute to cognitive impairment in these patients through grey matter damage or disconnections in essential cognitive regions^8,9^. The cumulative burden of these disconnections leads to cognitive decline, but a higher brain reserve may delay its onset despite severe disease burden^5,10^. Because brain reserve is potentially modifiable,^11^ early detection and intervention of triggers that disconnect brain networks are crucial for preserving brain reserve and preventing cognitive decline. Additionally, as patients may be unaware of cognitive decline in CIDDs, a sensitive and easily accessible screening tool is essential for monitoring cognitive function^12^.

The Symbol Digit Modalities Test (SDMT) is the predominant and widely accepted cognitive assessment tool for CIDDs^13–15^. This evaluation involves associating numbers with corresponding symbols within a 90 s timeframe, referencing a table with nine-digit-symbol pairs^16^. It is particularly effective for assessing processing speed and visual-spatial memory, domains prone to early decline in MS patients^17^. The National MS Society recommends early screening with the SDMT, followed by regular clinical assessments to monitor cognitive function^18^. Nevertheless, the SDMT has limitations. Beyond the inconvenience of employing pencil and paper, it lacks flexibility as participants must adhere to a fixed sequence of symbols and numbers in every trial. Furthermore, repeated testing with the same digit-symbol combinations can result in undesired learning effects^19,20^. By digitalizing the test and shortening its duration, the efficiency of the SDMT could be improved, rendering it more favorable and clinically useful for a wider patient population.

Recently, the processing speed test (PST) or CogEval, an iPad-based digital tool mimicking the SDMT, has been developed and shown effective in reflecting cognitive dysfunction and cerebral T2 lesion load in patients with MS has been demonstrated^21–23^. Additionally, several other digital tools for evaluating cognitive function or mood disorders in MS patients have also been developed and implemented^21,24–28^. However, these protocols maintained a fixed protocols without modifiable options, which offer the flexibility that is a strength of digital tools.

In this study, we developed and evaluated a modified digital symbol digit modality test (MD-SDMT) that incorporates diverse symbol-digit pairs in each trial, aiming to provide enhanced flexibility. We designed two distinct modifiable time protocols for the MD-SDMT, with test durations of 1 min (MD-SDMT_1 min) and 2 min (MD-SDMT_2 min). We aimed to assess the successful correlation of these MD-SDMT protocols with the gold standard paper-based SDMT and to investigate patients’ preferences concerning the different SDMTs, including the paper-based SDMT, MD-SDMT_1 min, and MD-SDMT_2 min.

## Results

### Baseline characteristics of the patients

**Figure 1:**
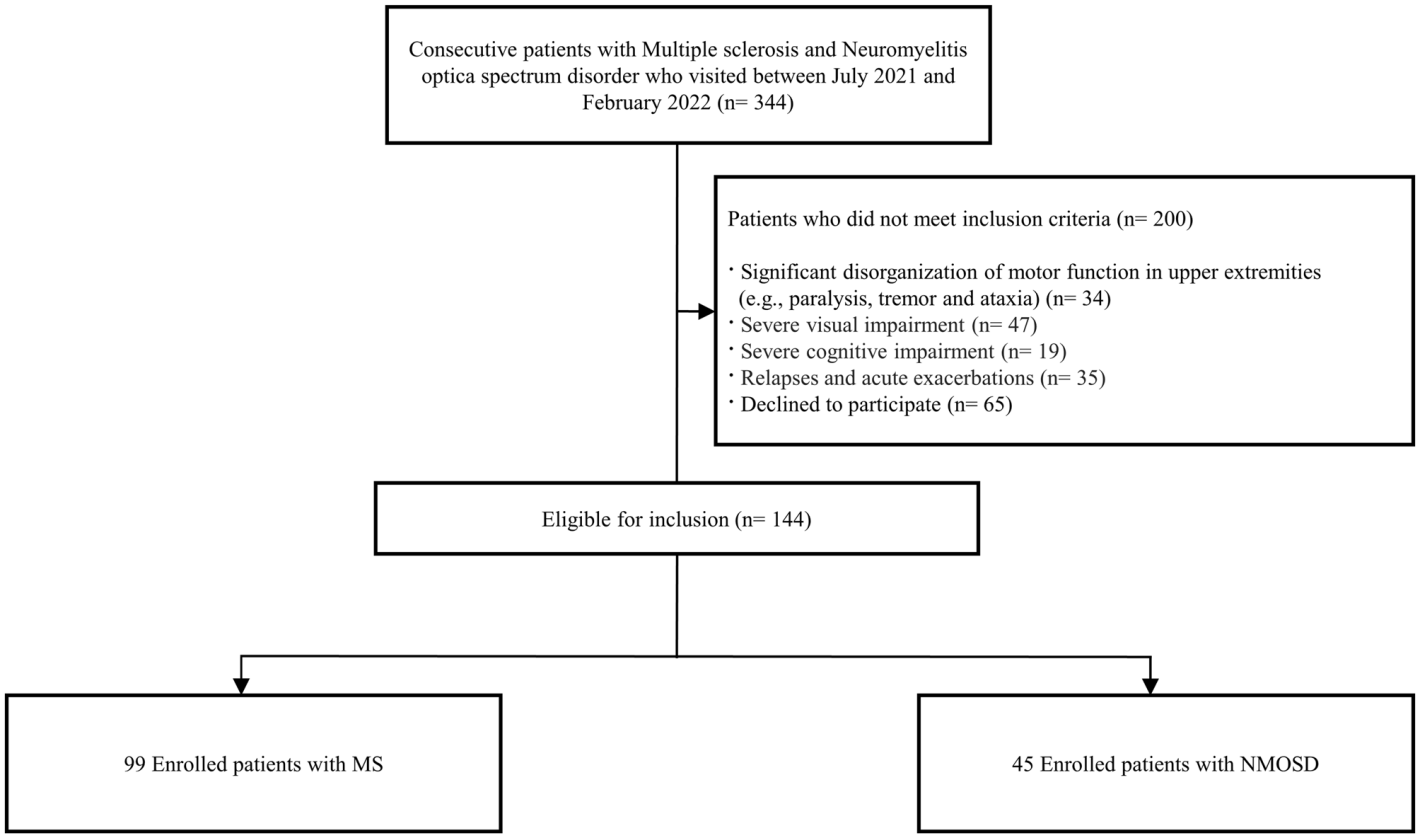
Flow chart of the patient selection criteria.

During the study period, 344 patients diagnosed with MS or NMOSD visited our center, and 144 (99 with MS and 45 with NMOSD) patients were enrolled (Fig. 1). The mean age was 49 years, with tercile values of 43, 57, and 76 years for the young-aged, middle-aged, and the old-aged tercile groups, respectively. Among the patients, 115 (79.9%) were women, and the median expanded disability status scale (EDSS) score was 2.0 (Table 1). The mean disease duration was 11 years, and the last clinical attack on enrollment occurred 4 years prior. The baseline mean paper-based SDMT score was 42.7. There were no significant differences in clinical variables between the MS and NMOSD groups, except for patient age, with the NMOSD group comprising older individuals. The degree of neurologic deficits was also comparable between the two groups.

**Table 1:** Baseline demographic and clinical characteristics of the patients.

	Total	MS	NMOSD	P*
	(n = 144)	(n = 99)	(n = 45)	
Age, y	50 (41–60)	48 (37–59)	53 (47–63)	0.023
Female	115 (79.9)	73 (73.7)	42 (93.3)	0.157
Education, y	14 (12–16)	14 (12–16)	12 (12–16)	0.113
EDSS score	2.0 (1.5–3.5)	2.0 (1.5–3.5)	2.5 (1.5–3.5)	0.275
Visual acuity				
Worse eye	0.6 (0.4–0.9)	0.6 (0.5–0.9)	0.6 (0.3–0.7)	0.007
Better eye	0.8 (0.6–1.0)	0.9 (0.6–1.0)	0.8 (0.5–0.9)	0.035
Disease duration, y	11 (5.3–15)	12 (6.5–15)	9.0 (5–16)	0.244
Disease course				
RRMS		96 (97.0)	N/A	
SPMS		2 (2.0)	N/A	
PPMS		1 (1.0)	N/A	
PRMS		0	N/A	
Interval from the last attack, y	4 (2–9.25)	4 (2–10)	3 (2–6)	0.202
Immunomodulatory treatment	128 (88.9)	83 (83.8)	45 (100)	0.157
Interferon-β		25 (30.1)	N/A	
Glatiramer acetate		1 (1.2)	N/A	
Teriflunomide		28 (33.7)	N/A	
Dimethyl fumarate		13 (15.7)	N/A	
Fingolimod		12 (14.5)	N/A	
Alemtuzumab		4 (4.8)	N/A	
Azathioprine		N/A	21 (46.7)	
Mycophenolate mofetil		N/A	11 (24.4)	
Rituximab		N/A	13 (28.9)	

Median (interquartile ranges) for continuous variables or number (percentage) for categorical variables.

MS: multiple sclerosis; NMOSD: neuromyelitis optica spectrum disorder; EDSS: expanded disability status scale; RRMS: relapsing–remitting MS; SPMS: secondary progressive MS; PPMS: primary progressive MS; PRMS: progressive relapsing MS; N/A: not applicable.

p*: MS vs. NMOSD.

### Paper-based SDMT and MD-SDMT scores

**Figure 2:**
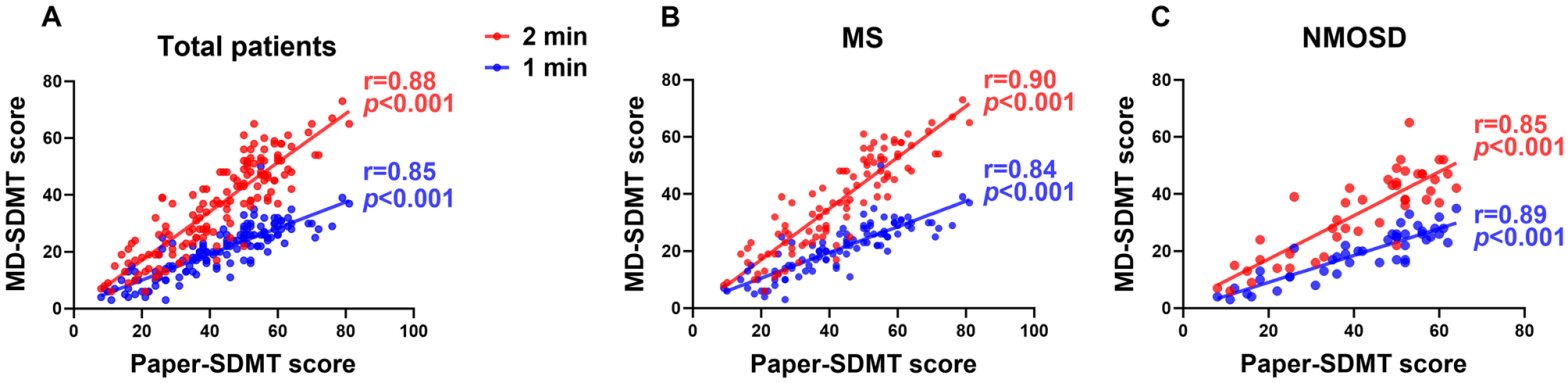
Correlations between scores from the MD-SDMT and paper-based SDMT. Scatterplots are presented comparing the performance of the MD-SDMT (1 min and 2 min protocols) versus paper-based SDMT administrations in patients with MS and NMOSD. All correlations are highly significant (p < 0.001). MD-SDMT, Modified Digital Digit Modalities Test; SDMT, Symbol Digit Modalities Test; r = , Pearson’s correlation statistic.

All participants successfully completed both MD-SDMT tests with different test time durations (one or two minutes). We evaluated the correlations between paper-based SDMT and MD-SDMT scores (Fig. 2 and Supplementary Table S1). Overall, in all participants, both MD-SDMT scores correlated well with paper-based SDMT scores (2 min, r = 0.88, p < 0.001; 1 min, r = 0.85, p < 0.001; p for interaction = 0.315)*.* These close correlations were comparable between patients with MS (2 min, r = 0.90, p < 0.001; 1 min, r = 0.84, p < 0.001; p for interaction = 0.082) and those with NMOSD (2 min, r = 0.85, p < 0.001; 1 min, r = 0.89, p < 0.001; p for interaction = 0.448).

**Figure 3:**
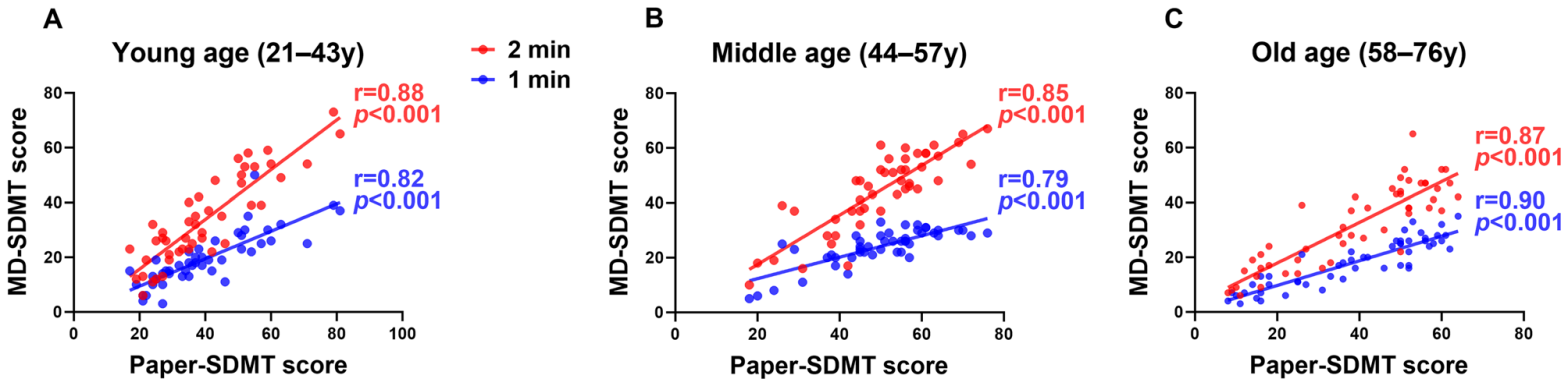
Correlations based on age groups between MD-SDMT (1 min and 2 min protocols) and paper-based SDMT performance. Age groups were classified into three categories based on participants’ age terciles: Young-, middle-, and old-age groups. All correlations are highly significant (p < 0.001). MD-SDMT, Modified Digital Digit Modalities Test; SDMT, Symbol Digit Modalities Test; r = , Pearson’s correlation statistic.

**Figure 4:**
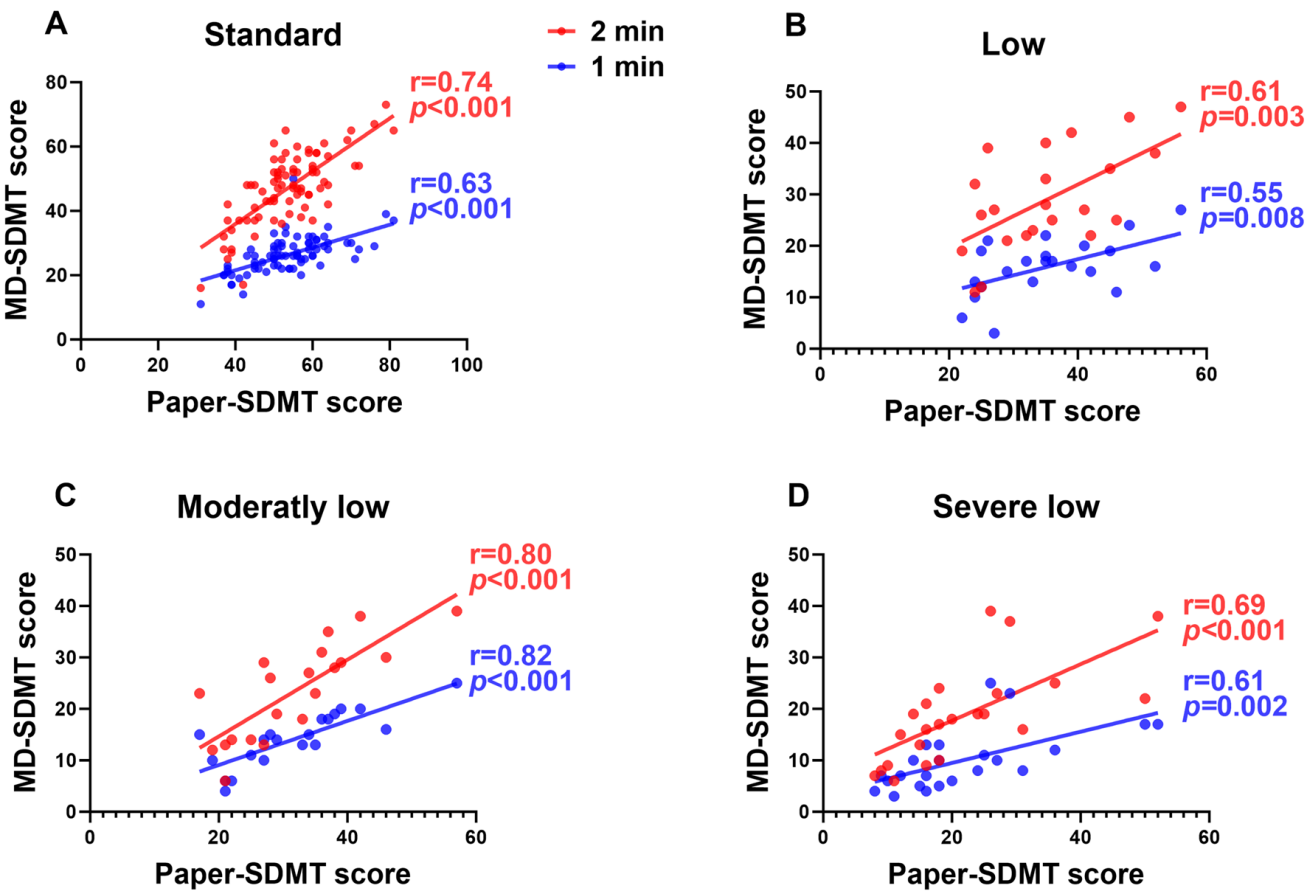
Correlations based on cognitive function groups between MD-SDMT (1 min and 2 min protocols) and paper-based SDMT performance. The degree of cognitive dysfunction was classified into four groups: standard, low, moderately low, and severe low, according to the SDMT score criteria. All correlations are highly significant (p < 0.01). MD-SDMT, Modified Digital Digit Modalities Test; SDMT, Symbol Digit Modalities Test; r = , Pearson’s correlation statistic.

We further analyzed correlations between paper-based SDMT and MD-SDMT scores according to the tercile age groups (the young-aged group; 21–43 years, the middle-aged group; 44–57 years, and the old-aged group; 58–76 years) (Fig. 3 and Supplementary Table S1). Regardless of the age group, the MD-SDMT scores exhibited a strong correlation with paper-based SDMT scores (young-aged: 2 min, r = 0.88, 1 min, r = 0.82, p for interaction = 0.310; middle-aged: 2 min, r = 0.85, 1 min, r = 0.79, p for interaction = 0.361; old-aged: 2 min, r = 0.87, 1 min, r = 0.90, p for interaction = 0.519). Additionally, we also examined correlations according to the paper-based SDMT performance, including the standard, low, moderately low, and severely low groups (Fig. 4 and Supplementary Table S1). To this end, MS patients were stratified into the standard (n = 60, 60.6%), low (n = 15, 15.0%), moderately low (n = 13, 13.1%), and severely low (n = 11, 11.1%) groups. NMOSD patients were categorized into standard (n = 19, 42.2%), low (n = 7, 15.5%), moderately low (n = 7, 15.5%), and severely low (n = 12, 26.6%) groups^13^. MD-SDMT scores from both protocols were significantly correlated with paper-based SDMT scores (standard group: 2 min, r = 0.74, 1 min r = 0.63, p for interaction = 0.198; low group: 2 min, r = 0.61, 1 min r = 0.55, p for interaction = 0.780; moderately low group: 2 min, r = 0.80, 1 min r = 0.82, p for interaction = 0.865; and severely low group: 2 min, r = 0.69, 1 min, r = 0.61, p = 0.660).

### Survey on patients’ experience

Among the participants, 120 individuals (83.3%; 86 with MS, 34 with NMOSD) provided consent and subsequently completed the questionnaire following the SDMT tests. The user experience survey primarily focused on the participants’ preferences among the tested SDMTs (paper-based SDMT, MD-SDMT_1 min, and MD-SDMT_2 min). Additionally, the survey sought insights into the patients’ perceived reliability of these tests. Regarding preferences for the assessment tools, the MD-SDMT_2 min was favored by 55% of patients, followed by the MD-SDMT_1 min (39.2%), and the paper-based SDMT (5.8%) (Table 2). Overall, participants exhibited a more positive response to both MD-SDMTs compared to the paper-based SDMT. Furthermore, the MD-SDMT_2 min was identified as the most reliable test by the majority (75%) of patients, followed by the MD-SDMT_1 min (28%), and the paper-based SDMT (2%).

**Table 2:** Patient responses regarding their preference and perceived reliability according to disease groups.

	Total	MS	NMOSD	p	p*
	(n = 120)	(n = 86)	(n = 34)		
The most preferred test				0.009	0.048
Paper-based SDMT	7 (5.8)	2 (2.3)	5 (14.7)		
MD-SDMT_1 min	47 (39.2)	31 (36.1)	16 (47.1)		
MD-SDMT_2 min	66 (55.0)	53 (61.6)	13 (38.2)		
The most reliable test				> 0.999	0.997
Paper-based SDMT	2 (1.7)	2 (2.3)	0		
MD-SDMT_1 min	28 (23.3)	20 (23.3)	8 (23.5)		
MD-SDMT_2 min	90 (75.0)	64 (74.4)	26 (76.5)		

For the categorical variables, data are presented as numbers (percentages).

MS: multiple sclerosis; NMOSD: neuromyelitis optica spectrum disorder; MD-SDMT: modified digital symbol digit modalities test; SDMT: symbol digit modalities test.

p* adjusted for age with the adjusted of covariance (ANCOVA).

When comparing the MS and NMOSD groups (Table 2), both groups showed a general tendency toward preferring the MD-SDMTs over the paper-based version. However, NMOSD patients exhibited a relatively higher preference for the paper-based SDMT compared to MS patients, and this difference was statistically significant (p = 0.009). Conversely, in terms of perceived reliability, there were no significant differences in patterns between MS and NMOSD patients (p > 0.999).

We further analyzed the results within age subgroups (Table 3). Across all age groups, more than half of the participants generally favored the MD-SDMT_2 min protocol over the other assessment tools. Notably, participants in the old-age group demonstrated a distinct preference for the paper-based SDMT, and were less inclined to favor the shorter, mobile device-based versions of the SDMT compared to the young- and middle-aged groups. Conversely, the young-aged group tended to prefer the MD-SDMT_1 min, the shorter version of the digital SDMT, than the other groups. This trend was statistically significant (p for trend = 0.027). The contrast in preferences was particularly evident when the responses from the old-aged group were compared with those from a combined dataset of the young- and middle-aged groups, as presented in Supplementary Table S2. Among patients with NMOSD, those who preferred the paper-based SDMT did not have worse visual function compared to those who favored the MD-SDMT (Supplementary Table S3). Regarding reliability, the MD-SDMT_2 min was consistently identified as the most reliable test, followed by the MD-SDMT_1 min and the paper-based SDMT; this trend remained consistent across all age groups. Notably, none of the participants in the old-age group identified the paper-based SDMT as the most reliable test.

**Table 3:** Patient responses regarding preferences and perceived reliability according to age groups.

	Young	Middle	Old	p	p for trend
	(n = 38)	(n = 43)	(n = 39)		
The most preferred test				0.178	0.027
Paper-based SDMT	1 (2.6)	1 (2.3)	5 (12.7)		
MD-SDMT_1 min	18 (47.4)	18 (41.9)	11 (28.3)		
MD-SDMT_2 min	19 (50)	24 (55.8)	23 (60)		
The most reliable test				0.420	0.653
Paper-based SDMT	2 (5.3)	0	0		
MD-SDMT_1 min	8 (21)	12 (27.9)	8 (20.5)		
MD-SDMT_2 min	28 (73.7)	31 (72.1)	31 (79.5)		

For the categorical variables, data are presented as numbers (percentages).

MD-SDMT: modified digital symbol digit modalities test; SDMT: symbol digit modalities test.

Finally, we analyzed the survey results according to the cognitive function of the patients (Table 4). Among the various cognitive function groups, the MD-SDMT_2 min consistently emerged as the most preferred and reliable assessment tool, surpassing other methods. Additionally, this preference remained consistent across all cognitive function groups, with no significant trends observed.

**Table 4:** Patient responses regarding preference and perceived reliability according to cognitive function groups.

	Standard	Low	Moderately low	Severely low	p	p for trend
	(n = 61)	(n = 20)	(n = 19)	(n = 20)		
The most preferred test					0.471	0.388
Paper-based SDMT	3 (4.9)	0	2 (10.5)	2 (10.5)		
MD-SDMT_1 min	28 (46)	8 (40)	5 (26.3)	5 (26.3)		
MD-SDMT_2 min	30 (49.1)	12 (60)	12 (63.2)	12 (60)		
The most reliable test					0.997	0.921
Paper-based SDMT	2 (3.3)	0	0	0		
MD-SDMT_1 min	15 (24.6)	5 (25)	4 (21.1)	4 (21.1)		
MD-SDMT_2 min	44 (72.1)	15 (75)	15 (78.9)	15 (78.9)		

For the categorical variables, data are presented as numbers (percentages).

MD-SDMT: modified digital symbol digit modalities test; SDMT: symbol digit modalities test.

## Discussion

This study demonstrates that MD-SDMT aligns closely with the gold-standard paper-based SDMT, providing reliable measurements across all participant groups. Patients generally favored the MD-SDMT over the paper-based SDMT. Although both the shorter (1 min) and longer (2 min) MD-SMDT durations demonstrated a significant correlation with the paper-based SDMT, there was a notable preference for the 2 min protocol. Notably, patients with NMOSD and those in the old-age group exhibited a greater preference for the paper-based SDMT, suggesting that individual preferences may vary based on patient characteristics.

The MD-SDMT demonstrated efficient assessment capabilities in clinical settings, showing a robust correlation with the paper-based SDMT across all participant groups, irrespective of patient characteristics including disease type (MS and NMOSD), age, and cognitive function levels. This is noteworthy, as the digital platform emerges as a potential substitute for the traditional paper-based SDMT in real-world clinical contexts. Furthermore, the 1 min and 2 min protocols of MD-SDMT are comparable in performance, indicating that reliable evaluations can be conducted in shorter test durations. This aligns with recent studies that found that the app-based SDMT requires only 46 s for adequate performance in patients with MS^24^. The varied test duration options enhance cognitive evaluation efficiency, particularly in time-constrained environments. Furthermore, the digital format offers the advantage of automated time tracking and scoring, thereby reducing the workload for examiners and providing immediate feedback to patients. This aspect of the MD-SDMT contributes to its high test–retest reliability, facilitating better periodic monitoring and more effective long-term data management.

Patient preferences for cognitive testing methods appear to be influenced by individual characteristics. Notably, patients with NMOSD and older individuals were more likely to prefer the paper based SDMT, as opposed to those with MS and younger individuals. Conversely, younger patients were more inclined towards the MD-SDMT_1 min, the shorter version of the digital test. The reason for the preference of the NMOSD group for the paper-based test over the MS group remains unclear. A possible factor may be age differences, as patients with NMOSD were significantly older. Generally, older patients who are more familiar with traditional methods (paper and pencils) tend to choose the paper based SDMT, while younger patients, who are accustomed to digital environments, prefer the shorter digital SDMT. Notably, despite their inclination for the paper-based test, no participant in the old-age group considered it the most reliable. Visual dysfunction, more severe in the NMOSD than in the MS group, did not significantly influence test preferences (Supplementary Table S3). Notably, despite their inclination for the paper-based test, no participant in the old-age group considered it the most reliable. Additionally, even among older individuals and the NMOSD group, only a minority favored the paper-based test over the MD-SDMT, indicating that digital cognitive tests are generally well-received by these patients.

The current study introduces several distinct features compared to previous studies (Supplementary Table S4). First, we have implemented a modifiable protocol in the MD-SDMT, demonstrating that patient preferences for test protocols can vary at the individual level. With the rapid advancement and application of digital technologies in clinical settings^29–36^, there is a growing emphasis on personalized care^37^. By adjusting aspects like task difficulty based on individual patient characteristics such as cognitive deficit levels more sensitive monitoring of cognitive function changes at the individual level becomes feasible. Moreover, allowing patients to choose their preferred testing protocol and establishing normative MD-SDMT scores for each protocol could enable continuous, personalized cognitive evaluations in the future.

Another noteworthy point of this study is that we evaluated the feasibility of a digital cognitive assessment tool for patients with NMOSD. Cognitive dysfunction in patients with NMOSD has received comparatively less attention, as neurodegeneration in this context is understood to be linked to relapses rather than progressive processes^38–40^. However, recent studies, including MRI findings, have revealed that patients with NMOSD also experience silent neurodegeneration similar to that observed in patients with MS^41–43^. Despite this, limited studies have been undertaken to investigate cognitive dysfunction in NMOSD^44–47^. Therefore, conducting longitudinal analyses using digital cognitive assessment tools can provide deeper insights into the cognitive profiles of NMOSD patients, enhancing our understanding of the underlying mechanisms and pathophysiology of cognitive dysfunction in this condition.

Finally, we evaluated patient responses, revealing that the MD-SDMT_2 min is the most preferred and reliable test. This preference suggests potential advantages of digital over paper-based SDMT, especially for adherence in managing chronic diseases^48^. Additionally, the preference for the MD-SDMT_2 min over the 1 min version across various age and cognitive function groups suggests that patients value ample testing time to fully demonstrate their abilities, rather than shorter evaluations.

This study has several limitations. First, due to its cross-sectional design, we did not investigate how the 1 min and 2 min MD-SDMT protocols correlate with the paper-based SDMT over time. Future longitudinal studies are necessary to assess if the performance of these protocols remains consistent over time, helping to establish the MD-SDMT’s effectiveness for monitoring long-term cognitive function. Second, our study focused exclusively on the written SDMT, excluding the verbal format. Consequently, patients with severe upper extremity impairments and NMOSD patients with major visual field impairments (20/200 vision or worse) were not included, as they were considered unable to participate in either protocol. This exclusion may have introduced selection bias. Third, the MD-SDMT primarily evaluates processing speed and does not assess other cognitive domains. While SDMT performance is associated with cognitive decline and disease progression in MS—considered the core common data element of cognition by the National Institute of Neurological Disorders and Stroke^14^—its utility in NMOSD patients is less certain^49^. Specifically, approximately 42% of NMOSD patients and 29% of MS patients identified as cognitively impaired by the brief cognitive screening test, which includes the SDMT from the Rao Brief Repeatable Neuropsychological Battery, were found to be cognitively normal when assessed with comprehensive tests such as the Wechsler Adult Intelligence Scale-III and the Wechsler Memory Scale-Revised^21^. Therefore, low performance in the MD-SDMT should not be hastily interpreted as true 'cognitive impairment,' especially in the NMOSD group. Fourth, the identical order of tests throughout the study may have influenced the results. Additionally, administering three consecutive tests within a short period may have led to mental fatigue, although no patients explicitly complained of mental fatigue during or after the test. Finally, we did not administer the MD-SDMT to a larger, more diverse group of patients or to the general population. The study population was small and not representative of the broader MS and NMOSD population, particularly those with progressive MS. Additionally, we were unable to calculate Z-scores that would standardize MD-SDMT scores across different populations. Future studies are needed to enhance the test’s applicability and generalizability.

## Conclusions

The MD-SDMT, whether in its 1 min or 2 min version, effectively aligns with the paper-based SDMT for assessing cognitive function in patients with MS and NMOSD. Participants demonstrated a stronger preference and perceived greater reliability for the longer MD-SDMT compared to both its shorter counterpart and the paper-based SDMT, although these preferences may differ based on individual patient characteristics.

## Methods

### Participants and inclusion criteria

Patients with CNS demyelinating diseases were prospectively enrolled from the outpatient clinic at the department of Neurology, Asan Medical Center (Seoul, South Korea) between July 2021 and January 2022. The inclusion criteria were as follows: (1) age $\ge$ 18 years; (2) provision of informed consent; (3) according to the diagnostic criteria or diagnosis of NMOSD with the presence of autoantibodies to aquaporin-4^50,51^; (4) being in remission for at least three months after a relapse event. In addition, the exclusion criteria were: (1) having a history of other medical illnesses that would affect neurological conditions, such as dementia and Parkinson’s disease; (2) having severe visual field impairment (e.g., 20/200 or worse); (3) having significant disorganization of motor function in upper extremities (e.g., paralysis, tremors, and ataxia); (4) being in a period of relapse or acute exacerbation. This study was approved by the hospital’s institutional review board (Asan Medical Center Institutional Review Board, IRB No. 2020–1038). This study was conducted in accordance with the Declaration of Helsinki. All participants provided informed consent prior to study initiation.

### Modified digital symbol digit modality test (MD-SDMT)

We developed the MD-SDMT, a tablet-based version of the SDMT, with two protocols modified from the original assessment. The modified protocols involved random key changes for nine symbol-digit pairs during each test trial, with test times set at 1 and 2 min as determined by the attending investigators. The symbols utilized were Unicode characters.

**Figure 5:**
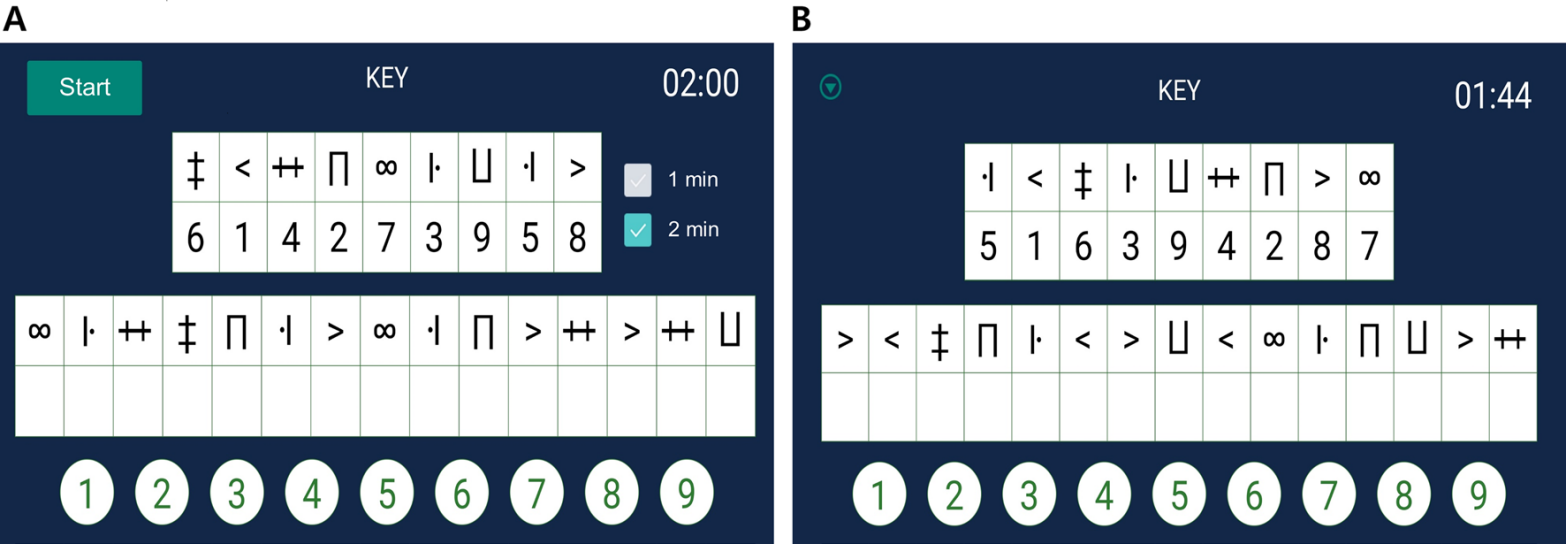
Illustration of the modified digital Symbol Digit Modalities Test (MD-SDMT). A (left): Screenshot of the tablet-based MD-SDMT during test administration. B (right): The key of nine symbol digits randomly changed during each trial. A new key is presented each time a line of response is completed. This procedure continues based on the test timeframe selected.

The top of the screen displayed ten symbol-digit pairs (Fig. 5). In the middle of the screen, 15 symbols without numbers were presented. Participants were requested to touch one of the numbers at the bottom of the screen to input the appropriate number corresponding to the symbol on the key. Each time the participant completed a row of tasks, consisting of 15 symbols, the symbol-digit pairs were randomized. This process continued until the test was terminated at either 60- or 120-s depending on the protocol. The SDMT app was operated on a tablet computer (Samsung Galaxy Tab S4), utilizing version 10 or higher of the Android operating system.

### Cognitive function assessments and preference survey

Under the supervision of a study investigator, participants underwent the MD-SDMT. Detailed instructions were provided to the patients to ensure that the test was correctly performed. During clinic visits, participants were evaluated sequentially with the paper-based SDMT, MD-SDMT_2 min, and MD-SDMT_1 min. For the paper based SDMT, participants were given a test paper and a pencil. The first line contained ten practice symbol-digit pairs, excluding the test time, and participants were requested to fill in the appropriate numbers below each symbol according to the key. Following the paper-based SDMT, participants typically started the MD-SDMT within 5 min.

Participants who agreed to undergo additional questionnaires provided feedback on their user experience with the assessment tools. The survey items investigated the preference of each patient and their thoughts on the reliability of the SDMT tests.

### Statistical analysis

Baseline characteristics were initially assessed using *t-*tests and chi-squared tests. We also compared the MD-SDMT_1 min, MD-SDMT_2 min, and paper-based SDMT according to the degree of cognitive dysfunction, patient age, and their preference by conditions using one-way analysis of variance. With regard to the degree of cognitive dysfunction, patients were classified into four groups: standard, low, moderately low, and severe low cognitive dysfunction groups according to the SDMT score criteria^13^. Age groups were classified into three categories based on participants’ age terciles. A linear–linear association was performed in the Chi-square test to investigate trends in preference and reliability of the MD-SDMT and paper-based SDMT across the age groups. Additionally, an analysis for trend was conducted by dividing the participants into young-, middle-, and old-age groups. Pearson correlation analyses were conducted to evaluate the associations between performances in the MD-SDMT_1 min (or MD-SDMT_2 min) and paper-based SDMT. Additionally, Fisher’s Z transformation was performed to compare the degree of these correlations. IBM SPSS Statistics software, version 21.0 (IBM, Armonk, NY, USA), was used to perform all statistical analyses. A *p*-value of < 0.05 was considered statistically significant.

### Supplementary Information

Supplementary Tables.

## Data Availability

Study data are available from the corresponding author upon a reasonable request.
