# Supplementary material for: Digital symbol-digit modalities test with modified flexible protocols in patients with CNS demyelinating diseases
Source: Sci Rep. 2024 Jun 25;14:14649. doi: 10.1038/s41598-024-65486-3 (PMC11199480; doi:10.1038/s41598-024-65486-3)
Supplement: Supplementary file 1 — Supplementary Tables. [file 41598_2024_65486_MOESM1_ESM.docx]

**Supplementary Table S1.** Correlations between MD-SDMT and paper-based SDMT

|  | **MD-SDMT-1min** | **MD-SDMT-2min** |  |
| --- | --- | --- | --- |
|  | **r_1_** | **r_2_** | p for interaction |
| Total Patients, (n = 144) | 0.85 | 0.88 | 0.315 |
| MS, (n = 99) | 0.84 | 0.9 | 0.082 |
| NMOSD, (n = 45) | 0.89 | 0.85 | 0.448 |
|  |  |  |  |
| Young age, (n = 46) | 0.82 | 0.88 | 0.310 |
| Middle age, (n = 52) | 0.79 | 0.85 | 0.361 |
| Old age, (n = 46) | 0.9 | 0.87 | 0.519 |
|  |  |  |  |
| Standard ,(n = 79) | 0.63 | 0.74 | 0.198 |
| Low, (n = 22) | 0.55 | 0.61 | 0.780 |
| Moderately low, (n = 20) | 0.82 | 0.8 | 0.865 |
| Severe low, (n = 23) | 0.61 | 0.69 | 0.660 |

r1: Pearson correlation coefficient of paper-SDMT and MD-SDMT-1min

r2: Pearson correlation coefficient of paper-SDMT and MD-SDMT-2min

z: Fisher's Z transformation of r1 and r2

**Supplementary Table S2.** Patient responses regarding the preference and perceived reliability according to binary age groups

|  | Young + Middle | Old |  |  |
| --- | --- | --- | --- | --- |
|  | (n =81) | (n = 39) | p | p for trend |
| The most preferred test |  |  | 0.036 | 0.020 |
| Paper-based SDMT | 2 (2.5) | 5 (12.8) |  |  |
| MD-SDMT_1 min | 36 (44.4) | 11 (28.2) |  |  |
| MD-SDMT_2 min | 43 (53.1) | 23 (59.0) |  |  |
|  |  |  |  |  |
| The most reliable test |  |  | 0.614 | 0.846 |
| Paper-based SDMT | 2 (2.5) | 0 |  |  |
| MD-SDMT_1 min | 20 (24.7) | 8 (20.5) |  |  |
| MD-SDMT_2 min | 59 (72.8) | 31 (79.5) |  |  |

For the categorical variables, data are presented as numbers (percentages).

MD-SDMT, modified digital symbol digit modalities test; SDMT, symbol digit modalities test

**Supplementary Table S3.** Visual function and preference in patients with NMOSD

|  | NMOSD  (n = 34) | EDSS | Visual function conversion score in EDSS | Worse eye | Better eye |
| --- | --- | --- | --- | --- | --- |
|  |  |  |  |  |  |
| The most preferred |  |  |  |  |  |
| Paper-based SDMT | 5 (14.7%) | 1.5 (0–2.5) | 1.0 (1.0–2.0) | 0.8 (0.0–1.3) | 0.9 (0.6–1.4) |
| MD-SDMT_1 min | 16 (47.1%) | 3.0 (1.6–3.5) | 2.0 (1.0–4.8) | 0.6 (0.1–0.8) | 0.8 (0.6–1.8) |
| MD-SDMT_2 min | 13 (38.2%) | 3.0 (2.3–6.5) | 3.0 (2.0–5.0) | 0.3 (0.0–0.6) | 0.6 (0.5–0.8) |

Median (interquartile ranges) for continuous variables or number (percentage) for categorical variables.

NMOSD, neuromyelitis optica spectrum disorder; EDSS, expanded disability status scale; MD-SDMT, modified digital symbol digit modalities test

**Supplementary Table S4.** Comparison of digital tools from previous studies

|  | **Rao SM, et al. CogEval** | **Pham, L. et al. NeuFun** | **Hsu W, et al. ACE** | **van Oirschot, et al. MS Sherpa** | **This study MD-SDMT** |
| --- | --- | --- | --- | --- | --- |
| **Participants** |  |  |  |  |  |
| MS | 165 | 154 | 53 | 25 | 99 |
| Age | 45 | 55 | 51 | 40 | 48 |
| Sex (female) | 52 | 89 | 17 | 23 | 73 |
| Disease duration | 11 | . | 12 | . | 12 |
| NMOSD | N/A | N/A | N/A | N/A | 45 |
| Age | . | . | . | . | 53 |
| Sex (female) | . | . | . | . | 42 |
| Disease duration | . | . | . | . | 9 |
| Healthy control | 217 | 39 | 24 | 79 | N/A |
|  |  |  |  |  |  |
| **Digital tool** |  |  |  |  |  |
| Format | Tablet | Smartphone | Tablet | Smartphone | Tablet |
| OS | iOS | Android | iOS | iOS/Android | Android |
| Evaluation | Processing speed | Processing speed | Processing speed | Processing speed | Processing speed |
| Symbol-digit key   matching sequence | Changing with every trial | Changing with every trial | N/A | Changing with every trial | Changing with every trial  as well as every row of response within a trial |
| Test time | Not modifiable | Not modifiable | Not modifiable | Not modifiable | Modifiable |
|  | (120 sec) | (90 sec) | (90 sec) | (90 sec) | (120 or 60 sec) |
|  |  |  |  |  |  |
| **Analysis** |  |  |  |  |  |
| Comparison with   Paper SDMT | Yes | Yes | Yes | Yes | Yes |
| Patients' feedback | N/A | N/A | N/A | N/A | Yes |
